# Plant-Made Trastuzumab (Herceptin) Inhibits HER2/Neu+ Cell Proliferation and Retards Tumor Growth

**DOI:** 10.1371/journal.pone.0017541

**Published:** 2011-03-03

**Authors:** Tatiana V. Komarova, Vyacheslav S. Kosorukov, Olga Y. Frolova, Igor V. Petrunia, Ksenia A. Skrypnik, Yuri Y. Gleba, Yuri L. Dorokhov

**Affiliations:** 1 A.N. Belozersky Institute of Physico-Chemical Biology, Moscow State University, Moscow, Russia; 2 N.N. Blokhin National Cancer Research Center, Russian Academy of Medical Sciences, Moscow, Russia; 3 N.I. Vavilov Institute of General Genetics, Russian Academy of Science, Moscow, Russia; 4 Nomad Bioscience GmbH, Biozentrum Halle, Halle (Saale), Germany; National Cancer Institute, United States of America

## Abstract

**Background:**

Plant biotechnology provides a valuable contribution to global health, in part because it can decrease the cost of pharmaceutical products. Breast cancer can now be successfully treated by a humanized monoclonal antibody (mAb), trastuzumab (Herceptin). A course of treatment, however, is expensive and requires repeated administrations of the mAb. Here we used an *Agrobacterium*-mediated transient expression system to produce trastuzumab in plant cells.

**Methodology/Principal Findings:**

We describe the cloning and expression of gene constructs in *Nicotiana benthamiana* plants using intron-optimized *Tobacco mosaic virus*- and *Potato virus X*-based vectors encoding, respectively, the heavy and light chains of trastuzumab. Full-size antibodies extracted and purified from plant tissues were tested for functionality and specificity by (i) binding to HER2/neu on the surface of a human mammary gland adenocarcinoma cell line, SK-BR-3, in fluorescence-activated cell sorting assay and (ii) testing the *in vitro* and *in vivo* inhibition of HER-2-expressing cancer cell proliferation. We show that plant-made trastuzumab (PMT) bound to the Her2/neu oncoprotein of SK-BR-3 cells and efficiently inhibited SK-BR-3 cell proliferation. Furthermore, mouse intraperitoneal PMT administration retarded the growth of xenografted tumors derived from human ovarian cancer SKOV3 Her2+ cells.

**Conclusions/Significance:**

We conclude that PMT is active in suppression of cell proliferation and tumor growth.

## Introduction

There was a time when most medicinal compounds were simply extracted from plants, but now, plant molecular biology produces valuable recombinant pharmaceutical molecules, including enzymes, vaccines, and antibodies [1]–[9]. Such “molecular farming” has many economic and qualitative benefits, including reduced health risks from human and animal pathogen contamination and comparatively high yields. It has been estimated that the cost of pharmaceutical protein production in plants could be 10- to 50-fold lower than production of the same protein in mammals [10], [11]. Plants rapidly accumulate single-chain [12]–[15] and full-size antibodies [16]–[20] and may produce personalised patient-specific anticancer vaccines [21]. Plants may be a source of biosimilars, new versions of known pharmaceuticals, including anticancer antibodies [22].

Human epidermal growth factor receptor 2 (HER2/neu) is an oncogene involved in abnormal cell growth in breast cancer and is a target for the humanised monoclonal antibody (mAb) trastuzumab (Herceptin) [23], which was approved by the US Food and Drug Administration for the treatment of HER2/neu-overexpressing breast tumours. HER2/neu is overexpressed in 20–30% of metastatic breast cancer patients where its overexpression results in the disruption of normal signaling pathways, causing the loss of cell growth regulation and the development of resistance to apoptosis. Trastuzumab induces antibody-dependent cellular cytotoxicity (ADCC), inhibits HER2-mediated signaling, and prevents cleavage of the extracellular domain of HER2 [24]. In HER2-positive breast cancer, trastuzumab has shown a survival advantage in early and metastatic disease and is now the standard of care [25]–[27]. Trastuzumab is produced by recombinant DNA technology in a mammalian cell (Chinese Hamster Ovary) culture. Recently, the production of plant-made trastuzumab [PMT] was shown in plant using the magnICON viral-based transient expression system [19]. Functional assays revealed that plant-produced trastuzumab and Herceptin have similar antiproliferative effects *in vitro* on HER2+ breast cancer cells.

Here, we used also genes encoding both heavy and light chains of trastuzumab, cloned into 35S- and virus-based vectors and expressed in *Nicotiana benthamiana* leaves. We show that both vector systems result in high yield of full-size antibodies, PMT, which recognizes HER2/neu on the surface of a human mammary gland adenocarcinoma cell line, SK-BR-3, and active in suppression of cell proliferation *in vitro*. Moreover, mouse PMT administration retarded efficiently the growth of xenografted Her2+ human ovarian tumors.

## Results

### Accumulation and purification of assembled PMT in *N. benthamiana* leaves

**Figure 1:**
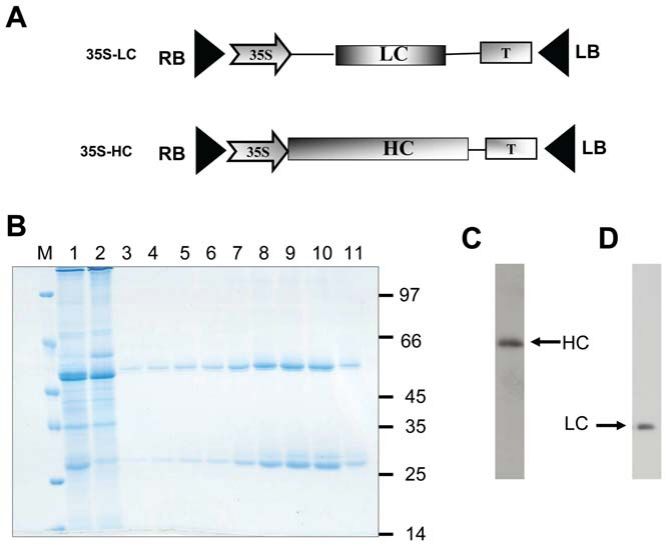
Production of assembled PMT in *N. benthamiana* leaves co-injected with 35S-based light- and heavy-chain-expressing vectors. **A** – Schematic representation of 35S-based light- (LC) and heavy-(HC) chain- expressing vectors 35S-LC and 35S-HC, respectively. 35S – Cauliflower mosaic virus 35S promoter, T – terminator of transcription, RB and LB – right and left borders from Ti-plasmid. **B** – Coomassie blue-stained SDS-PAGE proteins before purification (lane 1) and eluted fractions (3–11) obtained after protein A affinity chromatography, lane 2 – flow through after the first loading on the protein A column. M, molecular weight markers. **C**, **D** - Western blot analysis of PMT under reducing conditions, developed with anti-gamma (**C**) and -kappa (**D**)-chain-specific antibodies.

To prove the applicability of our plant transient system for the production of anticancer mAb, we synthesized genes encoding the heavy and light chains of the trastuzumab protein using the amino acid sequence published in DrugBank (accession number DB00072) and constructed 35S-based vectors (35S-LC and 35S-HC) (Figure 1A). *N. benthamiana* leaves co-agroinjected with PT-LC, PT-HC and the silencing suppressor *Tomato Bushy Stunt Virus* (TBSV) p19 [28] produced a high yield of PMT, as revealed in a gel stained with Coomassie blue. Assembled antibodies were extracted from plant tissue, purified on protein A affinity columns, and analyzed either by sodium dodecyl sulfate polyacrylamide gel electrophoresis (SDS-PAGE) under reducing conditions followed by Coomassie blue staining (Figure 1B) or by western blotting probed with gamma-HC- and kappa-LC-specific antibodies (Figure 1C,D). Bands corresponding to the heavy chain (∼55 kDa) and the light chain (∼25 kDa) are clearly visible on the Coomassie-stained gel (Figure 1B) and on western blots (Figure 1C, D). Expression of 35S-based constructs was maximal at 3 dpi, and the yield was between 100 and 150 µg/g of fresh weight (FW), depending on the experiment.

**Figure 2:**
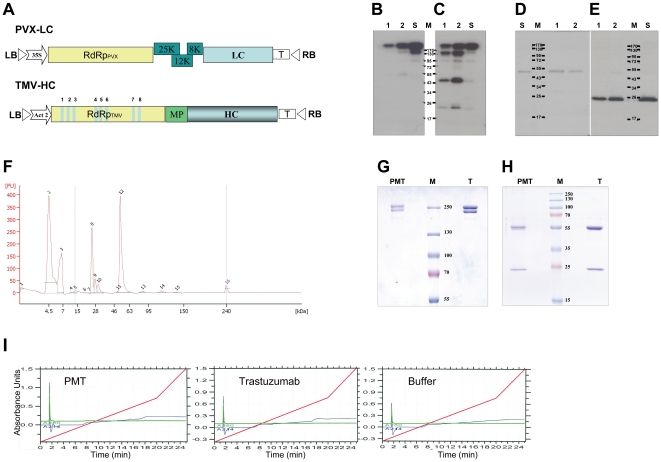
Accumulation and purification of assembled PMT in *N. benthamiana* leaves co-injected with light-chain-encoding PVX-based and heavy-chain-encoding TMV-based vectors. **A** – Schematic representation of PVX- and crTMV-based vectors. LB and RB, binary vector left and right borders, respectively; 35S, 35S promoter; Act 2, *Arabidopsis* actin 2 promoter; T, *nos* terminator; RdRp, RNA-dependent RNA polymerase; Bars 1–8, introns; MP, TMV movement protein; 25K, 12K, 8K, PVX movement protein genes. **B–E** - Western blot analysis of purified PMT. Purification of mAbs on protein A sepharose. Proteins were separated in a 10% polyacrylamide gel under non-reducing conditions (**B**, **C**) and in a 12% gel under reducing conditions (**D**, **E**) and transferred to a PVDF membrane. Western blots: **B** and **D** were probed with gamma-chain-specific antibodies; membranes **C** and **E** were incubated with kappa-chain-specific antibodies. 1–2, fractions from the protein A sepharose column; M, protein molecular weight markers; S, standard - 20 ng hIgG. **F** - Capillary electrophoresis analysis of PMT in reducing conditions on Agilent 2100 Bioanalyzer. Peak 12 corresponds to HC; peak 8 corresponds to LC. **G**, **H** – Comparison of PMT and trastuzumab. Proteins were separated in a 7.5% polyacrylamide gel under non-reducing conditions (**G**) and in a 12% gel under reducing conditions (**H**) and stained with Coomassie blue. **I** - RP-HPLC trace analysis of PMT and trastuzumab. The linear gradient was 0–60% acetonitrile for 20 min and then 60–100% acetonitrile for 5 min; the flow rate was 80 µL·min^−1^. The buffer blank was 10 mM Na-phosphate (pH 7.0). Absorbance at 214 nm and 280 nm is shown.

Next, PMT light and heavy chain genes were cloned into PVX-based and TMV-based vectors, respectively (Figure 2A), as these vectors are able to replicate within the same cell with high efficiency and do not compete with each other for replication binding sites [17]. Fully assembled PMT was extracted from *N. benthamiana* leaves co-injected with HC-TMV and LC-PVX vectors at 7 dpi when the maximal level of antibody production was detected (data not shown). Antibodies were purified on protein A sepharose columns and analyzed via SDS-PAGE under non-reducing (Figure S1A) or reducing (Figure S1B) conditions. MALDI-TOF analysis showed an identical peptide composition of PMT and trastuzumab light and heavy chains (data not shown). Assembled PMT is detected on gels stained with Coomassie blue. Western blot analysis was performed to determine the composition of the other bands on the gel. Probing with anti-gamma-chain antibodies revealed two high molecular weight bands, also detected with anti-kappa-chain antibodies (Figure 2B,C), that likely represent fully assembled IgG molecules and heterotrimers [(HC)_2_+LC]. The band that corresponds to the monomeric heavy chain is also visible in Figure 2B. Of these forms, the heterotetramer [(HC)_2_+(LC)_2_] is the most intense band visible after Coomassie blue staining (Figure S1B). Another band (∼95 kDa) detected on both 2B and 2C western blots appears to be a heterodimer of heavy and light chains. In addition, a strong band most likely corresponding to the dimeric form of the light chain (∼43 kDa) was produced with anti-kappa-chain antibodies. After treatment with 2-mercaptoethanol, all additional bands disappeared, with only heavy (Figure 2D) and light chains (Figure 2E) present. The yield of PMT expressed from viral vectors was between 200 and 300 µg/g FW depending on the experiment.

Further PMT purification on an AKTApurifier (GE Healthcare) was used to obtain assembled PMT that was free of additional complexes between heavy and light chains (Figure S2). Figure 2F shows capillary electrophoresis of PMT performed on an Agilent 2100 Bioanalyzer under reducing conditions, where peak 12 corresponds to HC and peak 8 corresponds to LC. It is likely that peaks 2 and 3 are low molecular products of PMT degradation.

Direct comparison of PMT and trastuzumab revealed a similar protein profile on gels stained with Coomassie blue (Figure 2G, H) and the absence of visible contaminations on HPLC trace analysis (Figure 2I).

### PMT recognises a HER2/neu peptide mimotope

**Figure 3:**
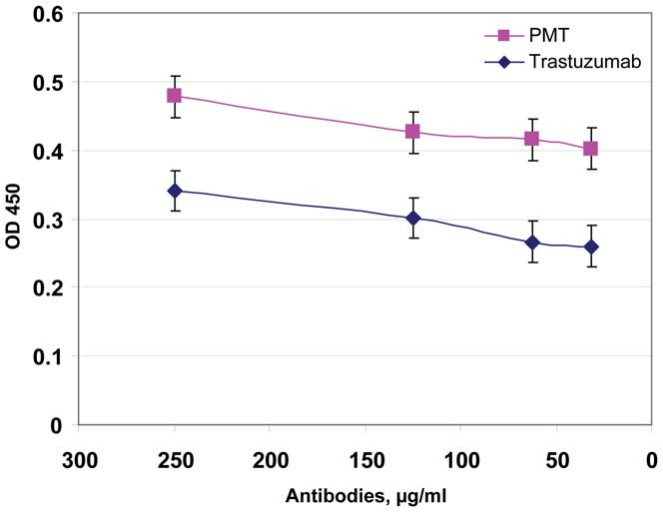
Binding of PMT to a HER2/neu peptide mimotope. Comparative binding of trastuzumab and PMT to the HER2/neu-specific cyclic synthetic peptide 563CYC (CHPECQPQNGSVTCFGPEADQCVACAHYKDPPFCVA) [30]. Microtiter wells were coated overnight with 2 µg/ml peptide and then blocked with 1% BSA for 1 h. The mAbs were then added to plates at a concentration of 250 µg/ml and serially diluted 1∶1 with phosphate buffered saline (PBS). Bound mAb was detected with HRP-conjugated anti-human IgG and then with substrate.

Trastuzumab binds amino acids 579 to 625 at the C-terminal end of domain IV of the extracellular region of HER2 [29]. Recently, the conformational epitope 563 to 598 of engineered trastuzumab demonstrated antitumour activity against HER-2/neu [30]. To examine whether PMT may bind the trastuzumab conformational epitope 563 to 598 we synthesised a cyclic synthetic peptide, 563CYC [29], [30] and compared PMT and trastuzumab binding by ELISA. Polystyrene plates were coated overnight with the 563CYC peptide and probed with PMT and trastuzumab the following day. Figure 3 shows that both mAbs, PMT and trastuzumab, bind the synthetic peptide 563CYC in a dose-dependent manner.

### PMT binds efficiently to HER2/neu-expressing SK-BR-3 cells

**Figure 4:**
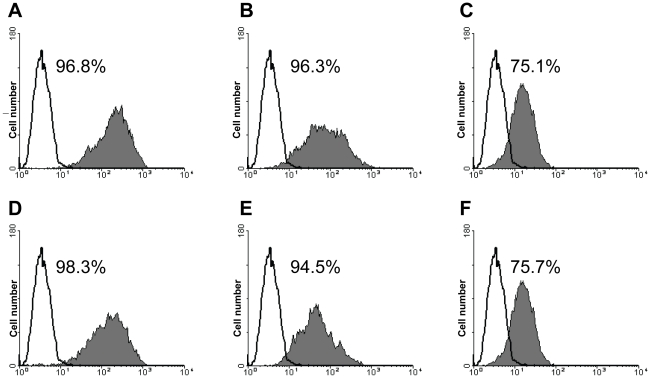
Examination of PMT binding to HER2/neu. Flow cytometry analysis of SK-BR-3 cells expressing HER2/neu incubated with trastuzumab (A–C) and PMT (D–F) in the following concentrations: 10 µg/ml (A,D), 1 µg/ml (B,E), and 0.1 µg/ml (C,F). Cells incubated only with secondary reagents were included as a control (open peak). Shadowed areas show specific binding. The percentage of cell surface expression of HER2/neu in SK-BR-3 cells is shown. These data represent three separate experiments.

For quantitative estimation of the binding affinity of PMT to Her2/neu antigen displayed on cells, FACS analysis was performed. Figure 4 (D–F) shows a high percentage (75.7% to 98.3%) of PMT binding to surface HER2/neu independently of antibody concentration. This result is similar to the data obtained using trastuzumab (Figure 4A–C).

Next, immunocytochemical staining of a human mammary gland adenocarcinoma cell line that overexpresses HER2/neu, SK-BR-3, was performed to test the functional activity of the plant-made mAb. PMT bound to Her2/neu oncoprotein on the surface of these cells as effectively as the diagnostic antibody A0485 (Dako, Denmark) (data not shown). The same result was obtained on tissue samples from a patient with Her2/neu-positive cancer (data not shown).

We conclude that PMT and trastuzumab exhibit no difference in binding capacity for HER2/neu.

### PMT inhibits SK-BR-3 cell growth *in vitro*

**Figure 5:**
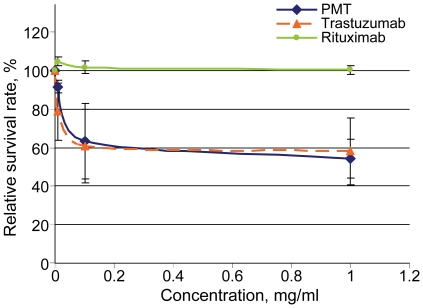
Effects of PMT on growth of the breast cancer cell line SK-BR-3 in MTT assays. Growth inhibition effect of PMT compared to trastuzumab (Herceptin, Hoffmann-La Roche) and rituximab (Hoffmann-La Roche) as a negative control. This assay was repeated at least three times.

The SK-BR-3 cell line was used to compare the antiproliferative properties of PMT and trastuzumab. Varying concentrations of PMT (0.1–1.0 mg/ml) were added to cell cultures, and their effects on cell growth were assessed in triplicate MTT assays. The data presented in Figure 5 show similar inhibitory effects of PMT and trastuzumab on SK-BR-3 cell proliferation. We conclude that PMT possesses the anticancer properties of trastuzumab.

### PMT retards SKOV3-derived tumor growth in a xenograft mouse model of human ovarian cancer

Having shown that PMT suppresses tumor cell growth, we investigated its antitumor effects in SKOV3 Her2+ cells implanted into mice. Although it is known that SKOV3-derived tumors are less sensitive to trastuzumab than are SK-BR-3-derived tumors [31], this model reveals the antitumor activity of PMT.

**Figure 6:**
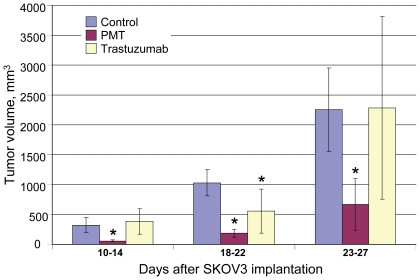
PMT inhibits tumor growth in a xenograft model of human Her2+ ovarian cancer. The treatment groups received their first doses (20 mg/kg) of PMT (n = 7) and trastuzumab (n = 10) in saline solution i.p. 6 days after SKOV3 implantation, and then for 16 days, they received 8 consecutive injections (10 mg/kg). The control group (n = 34) received saline solution. Tumor volumes were recorded in intervals 10–14, 18–22 and 23–27 days after SKOV3 implantation using a caliper. Data are the mean ± standard deviations from two independent experiments. Asterisk shows P<0.05 by the unpaired two tailed Student's t-test for statistical significance of difference between the PMT and trastuzumab treatment and control.

As shown in Figure 6, PMT treatment caused a delay in tumor growth. After 8 consecutive injections (10 mg/kg, see Material and Methods), the reduction in tumor growth was 70% compared to control mice treated with saline solution. Ten days after the last administration of PMT, there was an overall 80% reduction in tumor growth. Trastuzumab injections demonstrated a low effect on tumor growth.

We conclude that PMT possesses the major antitumor activity.

## Discussion

There are two strategies of antibody engineering for plant production [32]. First, one can employ antibody in miniaturized format based on the observation that H chains retain some antigen-binding capacity even in the absence of L chains. The smallest format found to keep full binding activity is the so-called single-chain fragment (scFv), in which the two variable regions, VH and VL, are artificially linked by a flexible polypeptide [12]–[15]. The other miniaturized type is the H-chain antibody derived from camelids (camels and llamas) where the L chain is missing [33]. These miniaturized antibodies do not require glycosylation and can be assembled in both plant and prokaryotic systems such as *Escherichia coli*. For example, scFv-based antibodies against HER2/neu have been produced in *E. coli* and in plants using both stable and transient systems in tobacco and *Nicotiana benthamiana* [12]–[15].

The second strategy is the creation of full-sized antibodies because of their widespread use as anti-tumor agents [16]–[19] and the fact that plants exhibit a similar endomembrane system and secretory pathway compared to human cells [34]. Although protein glycosylation in plant cells is slightly different from that of animal cells [35], “humanized” *N. benthamiana*, Arabidopsis thaliana, and *Lemna minor* plant lines have been generated [36]–[38]. Many different forms of full-sized antibodies have been produced in plant systems using either transient expression systems or stable transgenic plants [16]–[20]. The latter strategy suffers from generally low protein yields. In contrast, plant viral vectors demonstrate a high potential to rapidly produce full-size antibodies. In 2006, Giritch *et al.* [17] developed virus-based transient expression approaches (magnICON system) [39]–[42] for scalable production of full-size anticancer mAbs, creating an opportunity for plant-made pharmaceuticals [22]. The above system exploits pro-viral vectors and intron optimization of the TMV vector in which putative cryptic splice sites were removed and multiple plant introns were inserted [43], [44]. Full mAb production requires simultaneous expression of light- and heavy-chain-encoding genes in the same plant cell infected with two different non-competing viruses, such as TMV and PVX [17].

Here, we used 35S-based vectors and an assembled viral vector system in which intron-optimized TMV and PVX vectors encoded the heavy and light chains of PMT, respectively. Both viral and non-viral systems directed production of PMT in *N. benthamiana* leaves; however, high antibody production (100 to 150 µg/g FW) from non-replicating vectors can be achieved only after TBSV p19 co-injection (Figure 1). Joint injection of TMV and PVX vectors provided a yield of purified antibody up to 300 µg/g FW and excluded the requirement of adding an anti-silencing gene into the inoculation mixture.

Herceptin (trastuzumab) is a humanized mouse monoclonal antibody 4D5 and binds to the domain IV of HER2 [29]. The precise mechanisms underlying its action and acquired resistance are still poorly understood. Recent studies have shown that Herceptin does not decrease HER2 phosphorylation [45], [46]. This failure to abolish HER2 phosphorylation may explain why acquired resistance inevitably occurs for all patients if Herceptin is given as monotherapy [47].

Recently, Grohs et al. [19] used the magnICON system and demonstrated that PMT produced in *N. benthamiana* inhibited the growth of HER2-positive cancer cells. Functional assays revealed that PMT and Herceptin have similar *in vitro* antiproliferative effects on breast cancer cells that overexpress HER2. Here in line with results of Grohs et al. [19], our experiments showed that PMT efficiently suppressed SK-BR-3 cell growth i*n vitro*. Moreover, our purified PMT was as robust as trastuzumab in recognizing the HER2/neu peptide mimotope (Figure 3) and HER2/neu oncoprotein on the surface SK-BR-3 cells (Figure 4).

Surface plasmon resonance (SPR) spectroscopy is a potential technique for the affinity profile identification of the molecules. Although first SPR studies for trastuzumab were inconclusive [48], further experimentation is needed to compare binding of these antibodies to both the HER2/neu antigen and Fcγ receptor.

Our direct testing of antitumor activity showed that PMT efficiently retarded the growth of xenografted tumors derived from human ovarian cancer SKOV3 Her2+ cells (Figure 6). Additionally, PMT turned out to be more effective than trastuzumab in suppression of tumor growth. The cause of this phenomenon is unclear. SPR study may reveal differences in the affinity of trastuzumab and PMT to the antigen and/or Fcγ receptor, which may help explain the enhanced tumour-restricting properties of PMT *in vivo*. We suggest that it is too early to claim that PMT is biosimilar to trastuzumab. Additional experiments are required to prove that trastuzumab and PMT share full identity in their amino acid sequence, glycosylation profile and ADCC.

## Materials and Methods

### Gene and vector engineering

The trastuzumab amino acid sequence (DrugBank accession number DB00072) was used to synthesise the PMT light (LC) and heavy (HC) chain genes. The Enthelechon backtranslation tool (Markus Fischer, Backtranslation Tool, http://www.entelechon.com/backtranslation, Entelechon GmbH, Regensburg, Germany) was used for codon sequence determination.

35S-based vectors (35S-LC and 35S-HC) were made by replacing the GFP-RFP cassette with LC or HC genes in a 35S-GFP-RFP vector [49] using NcoI-XhoI sites.

The TMV-based vector was made in several cloning steps with intermediate construct (IC) formation. A single, 1283 nucleotide EcoRI-BamHI fragment from pICH4351 [43] was inserted into pGEM3Z to create IC-1. To produce IC-2, two oligonucleotides (“pl+” TCGACAGCTAGCTCCATGGACTCGAGT and “pl−” GTACACTCGAGTCCATGGAGCTAGCTG) were annealed and inserted into IC-1 using XhoI-BsrGI sites. Next, the HC gene was cloned into the IC-2 digested with NcoI-XhoI, resulting in IC-3. In the final cloning step, crucifer infected TMV (crTMV)-based vector with coat protein (CP) fused with GFP gene (crTMV-CP-GFP) [50] was digested with KpnI-BHI and used as a vector; the first fragment contained the *Arabidopsis* actin 2 promoter. The TMV RNA-dependent RNA polymerase (RdRp) with eight introns and a part of the TMV movement protein gene were obtained from pICH17388 (courtesy of Icon Genetics GmbH, and described in Giritch *et al.*, 2006) [17] and flanked with KpnI and EcoRI sites. IC-3 was digested with EcoRI and BamHI, resulting in the second fragment. The TMV-HC vector was achieved by ligation of these fragments.

Vector PVX-LC was made by modifying PVX-BIN19 [51]; the CP gene was replaced with the LC coding sequence.

### Agroinfiltration

*Agrobacterium tumefaciens* strain GV3101 was transformed with individual binary constructs and grown at 28°C in LB medium supplemented with rifampicin 50 mg/L, gentamycin 25 mg/L and either carbencillin 50 mg/L or kanamycin 50 mg/L. An aliquot of *Agrobacterium* cell suspension from an overnight culture (2 ml) was diluted in 10 mM MES buffer (pH 5.5) supplemented with 10 mM MgSO_4_ to a final OD_600_ of 0.3. Agroinfiltration was performed on almost-fully-expanded *N. benthamiana* leaves still attached to the intact plant. A bacterial suspension was infiltrated into the leaf tissue using a 2-ml syringe, after which the plants were grown under greenhouse conditions at 22°C with 16 hours of light.

### PMT extraction and purification

Total soluble protein was extracted from agroinoculated *N. benthamiana* leaves with 10 mM sodium phosphate buffer. PMT isolation from crude plant extract was performed with either Protein A Sepharose™ 4 Fast Flow (GE Healthcare) or 1 ml HiTrap Protein A HP columns (GE Healthcare) according to manufacturer's protocol. A “Sartobind Q nano” membrane (Sartorius Stedim Biotech) was used for further purification to remove viruses, DNA and endotoxins.

### HPLC equipment and conditions

HPLC analyses were performed on a narrow-bore column (Milichrom A-02; EnviroChrom LC, Chromatography Institute ECONOVA, Novosibirsk, Russia; 75×2 mm) packed with 5-lmparticles of Nucleosil C18, pore size 120 Å (Macherey-Nagel, Duren, Germany). Separations were performed at 25°C, and a dual wavelength (214 nm and 280 nm) detector was used. The elution gradient profile was as follows. The elution solvents were A (0.1% trifluoroacetic acid in water, pH 2.2) and B (acetonitrile with 0.1% trifluoroacetic acid). The linear gradient was 0–60% B in 20 min and then 60–100% B in 5 min; the flow rate was 80 µL·min^−1^. Fractions were collected for subsequent analysis using a Gilson 201 fraction collector.

### SDS-PAGE, western blot analysis and ELISA

Samples (15 mg) of agroinfiltrated *N. benthamiana* leaves were ground in the presence of celite in 50 µl of PBS. Crude leaf extracts were resolved on 7.5 to 10% (non-reducing conditions) or 12% (reducing conditions) polyacrylamide gels using Laemmli's buffer system [52] followed by Coomassie brilliant blue G-250 staining. For western blot analysis, fractionated proteins were transferred to a Hybond-P PVDF membrane (GE Healthcare), blocked with 5% skim milk (Fluka) in TBS and probed with goat human-kappa-chain-specific HRP-conjugated antibodies (Sigma) or goat human-gamma-chain-specific HRP-conjugated antibodies (Sigma) diluted 1∶15,000 in TBS with 0.1% Tween 20. The western blot was developed with an ECL detection reagent (GE Healthcare). The ELISA procedure was described earlier [53].

### Cell Proliferation Assay

The effect of anti-HER-2/neu mAbs on proliferation of the human mammary adenocarcinoma cell line SK-BR-3 was investigated by the MTT [3-(4,5-Dimethylthiazol-2-yl)-2,5-iphenyltetrazolium bromide] assay as described [54], using saturating mAb concentrations. Cells (1×10^4^ cells per well) were seeded in 96-well plates. After exposure to the different drugs for 48 h, 20 mL of MTT solution (5 mg/mL in PBS) was added to each well, and the plates were incubated for an additional 4 h at 37°C. The MTT solution in the medium was removed by aspiration. To achieve solubilization of the formazan crystal formed in viable cells, 150 mL of dimethylsulfoxide (DMSO) was added to each well before absorbances (A) at 570 nm were measured. Cell survival was calculated as the ratio of A_570_ nm in wells containing a PMT compared to that in control wells with no PMT.

### Nude mouse xenograft model of HER2+ SKOV3-derived human ovarian cancer

This study was carried out in strict accordance with the recommendations in the Guide for the Care and Use of Laboratory Animals of the N. N. Blokhin National Cancer Research Center, Moscow, Russia. The protocol was approved by the Committee on the Ethics of Animal Experiments of the N. N. Blokhin National Cancer Research Center, Moscow, Russia (Permit Number: 22, May 18, 2009). All surgery was performed under sodium pentobarbital anesthesia, and all efforts were made to minimize suffering.

Five million SKOV3 cells were s.c. injected into 4- to 6-wk-old female BALB/athymic nude mice (Animal Center of N. N. Blokhin National Cancer Research Center, Moscow, Russia). Six days after SKOV3 implantation, when the average tumor volume was 41±15.5 mm^3^, the treatment groups received their first dose of PMT (20 mg/kg). Then for 16 days, the mice received 8 consecutive injections (10 mg/kg). The control group received normal saline solution. Tumor volumes were recorded using a caliper 10, 14, 18, 22, 23 and 27 days after SKOV3 implantation.

## Supporting Information

Figure S1 **Purification of PMT using protein A sepharose.** Proteins were separated in an 8% polyacrylamide gel under non-reducing conditions (**A**) and in a 10% gel under reducing conditions (**B**) and stained with Coomassie blue. Lanes **1–7**, fractions from the protein A sepharose column; lane 8, flow through from the column; lane **M**, protein molecular weight markers; lane **S**, standard - 1 µg hIgG.
(TIF)

Figure S2 **Further PMT purification.** **H** - PMT was purified on an AKTApurifier (GE Healthcare) using 1 ml HiTrap Protein A columns. Lanes 1–10, fractions from the protein A sepharose column; lane 11, Sartobind Q nano purified protein. Protein eluted from Sartobind with 1 M NaCl – lane 12.
(TIF)
