# Supplementary figures and images for: Plant-Made Trastuzumab (Herceptin) Inhibits HER2/Neu+ Cell Proliferation and Retards Tumor Growth
Source: PLoS One. 2011 Mar 3;6(3):e17541. doi: 10.1371/journal.pone.0017541 (PMC3048398; doi:10.1371/journal.pone.0017541)

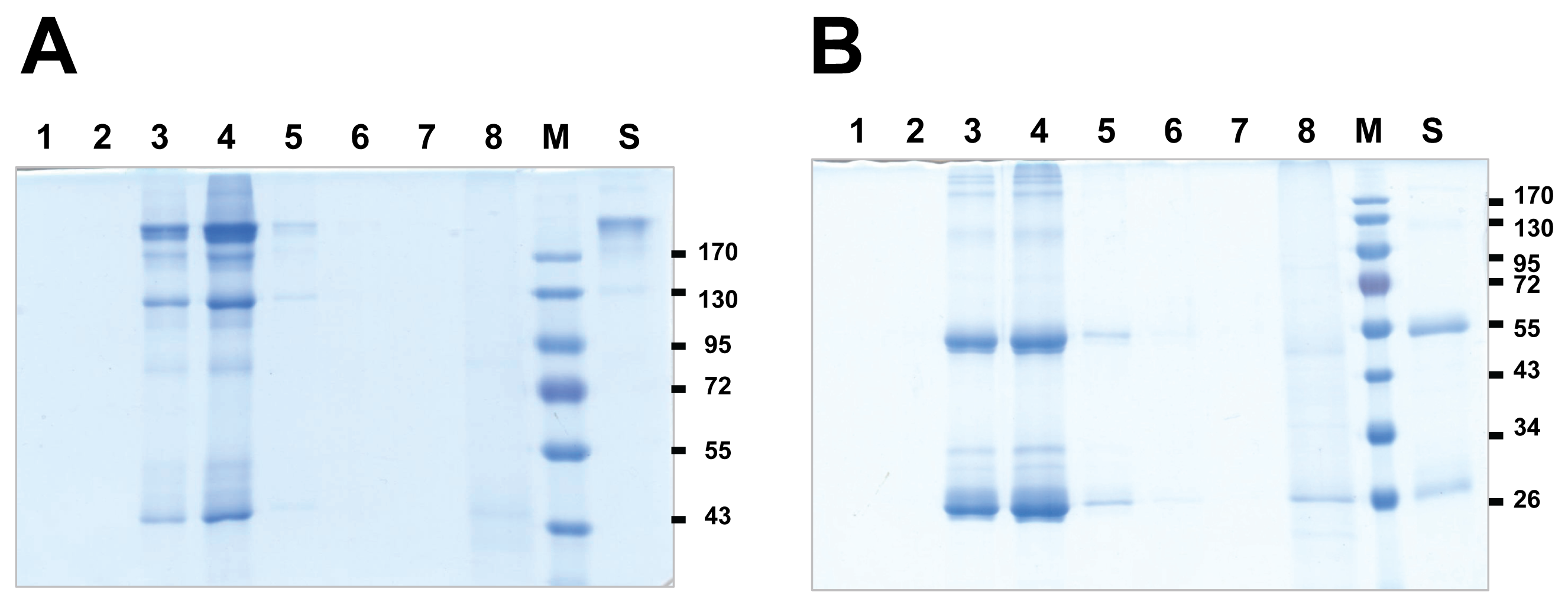

Supplement: Figure S1 — Purification of PMT using protein A sepharose. Proteins were separated in an 8% polyacrylamide gel under non-reducing conditions (A) and in a 10% gel under reducing conditions (B) and stained with Coomassie blue. Lanes 1–7, fractions from the protein A sepharose column; lane 8, flow through from the column; lane M, protein molecular weight markers; lane S, standard - 1 µg hIgG. (TIF) [file pone.0017541.s001.tif]

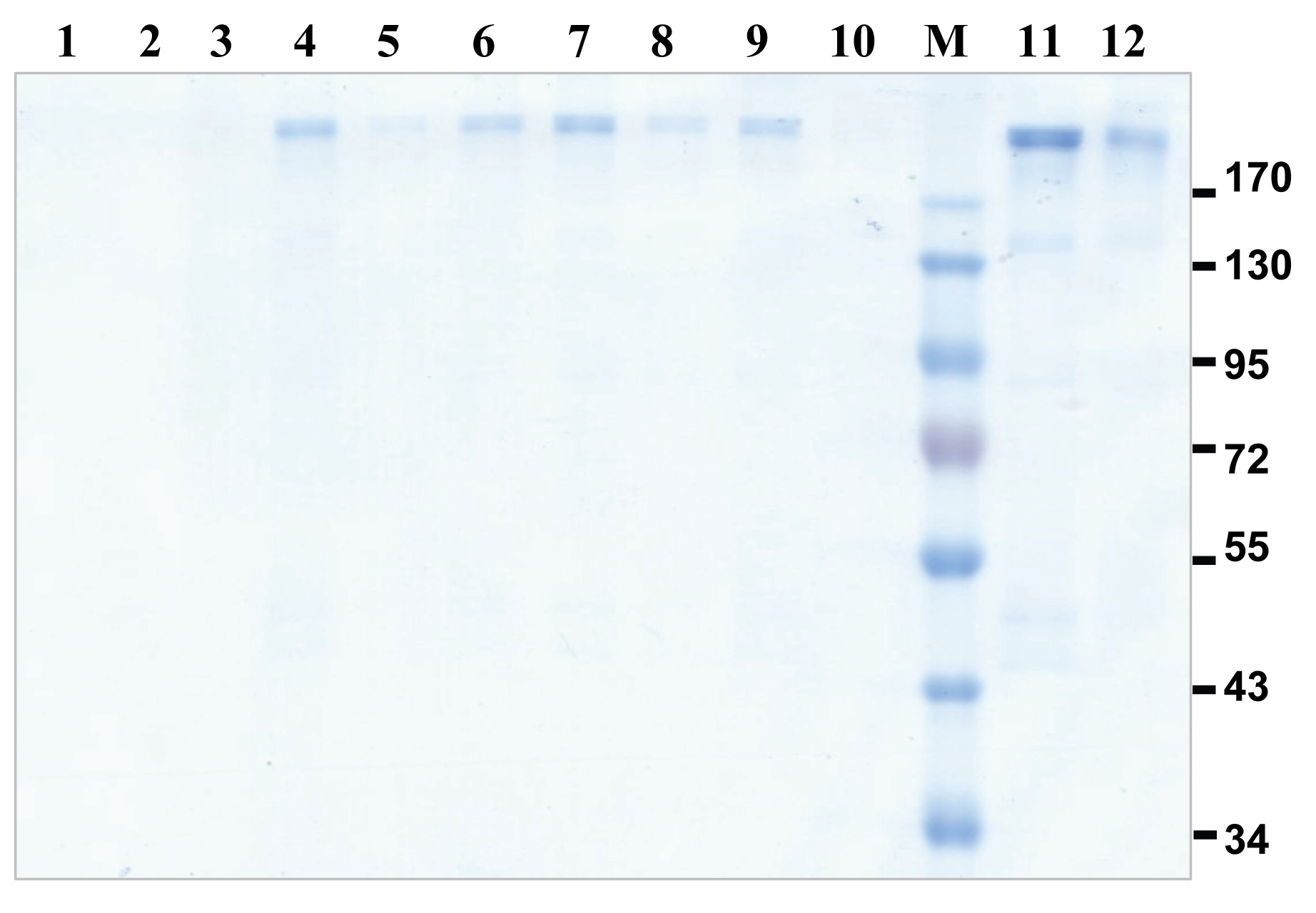

Supplement: Figure S2 — Further PMT purification. H - PMT was purified on an AKTApurifier (GE Healthcare) using 1 ml HiTrap Protein A columns. Lanes 1–10, fractions from the protein A sepharose column; lane 11, Sartobind Q nano purified protein. Protein eluted from Sartobind with 1 M NaCl – lane 12. (TIF) [file pone.0017541.s002.tif]
